# Cancer of the Breast Treated by Removal of Ovaries

**Published:** 1902-10-11

**Authors:** 


### CANCER OF THE BREAST TREATED BY REMOVAL OF OVARIES.

Mr. D'Arcy Power [1] records three cases in which he removed the ovaries in patients with inoperable carcinoma of the breast. Two of the patients had passed the menopause, and in neither of them was the operation followed by any improvement; but in the case of the woman in whom the change of life had not occurred the removal of the ovaries was followed by an arrest and apparently a retrocession of the cancerous process. As showing how curiously, nay we may say how mysteriously, removal of the ovaries seems sometimes to influence the cancer process in the breast, and as suggesting that the progress or retrogression of a carcinoma hinges upon some comparatively slight change in the nutritive processes in the part involved, the details of this case are of much interest. The patient was a widow, aged 42 years, who was admitted into St. Bartholomew's Hospital on April 21, 1902. She was suffering from a carcinoma of the right breast which she had noticed for a year. The tumour was a large one; the breast was immovably fixed to the deeper tissues; there was also a suspicious swelling in the left breast, and there were secondary growths in the skin, the axillary glands, and the infra-clavicular fossa. It was unanimously agreed that the case was inoperable by the ordinary methods. The ovaries on their removal proved to be cystic and contained nodules of spheroidal-celled carcinoma. A month later the patient left the hospital. After the operation there had been much pain in both breasts, but at the time of her discharge they were painless, the growth in the right breast was smaller and softer, whilst the nodules in the skin were much less marked than at the time of her admission. On presenting herself again a month later, that is in June, she was much improved in health, menstruation which had been regular up to the operation had ceased, the left breast was nearly free from mastitis, the right breast was freely movable, and the skin over it much less nodular, the growth in the breast was smaller, and the enlarged glands were diminished but had by no means disappeared. At the end of August the patient was still in good health and her colour was much improved; the right breast was even more soft and movable than at the last visit, and the glands in the axilla were imperceptible. The left breast still showed signs of mastitis and there was pain along the inner side of the left arm. There can be no doubt then that the operation had produced a distinct effect upon the progress not only of the growth but of the glands.

[1] Lancet, Oct. 4.

